# Opposing effects of dietary n-3 and n-6 fatty acids on mammary carcinogenesis: The Singapore Chinese Health Study

**DOI:** 10.1038/sj.bjc.6601340

**Published:** 2003-10-28

**Authors:** M Gago-Dominguez, J-M Yuan, C-L Sun, H-P Lee, M C Yu

**Affiliations:** 1 USC/Norris Comprehensive Cancer Center, Keck School of Medicine of the University of Southern California, 1441 Eastlake Avenue, Los Angeles, CA 90089-9181, USA; 2 Department of Community, Occupational and Family Medicine, National University of Singapore, Singapore

**Keywords:** fat, polyunsaturated fatty acids, n-3 fatty acids, n-6 fatty acids, fish, breast cancer, Chinese

## Abstract

We investigated the effects of individual fatty acids on breast cancer in a prospective study of 35 298 Singapore Chinese women aged 45–74 years, who were enrolled during April 1993 to December 1998 (The Singapore Chinese Health Study). At recruitment, each study subject was administered, in-person, a validated, semiquantitative food frequency questionnaire consisting of 165 food and beverage items. As of December 31, 2000, 314 incident cases of breast cancer had occurred. We used the Cox regression methods to examine individual fatty acids in relation to breast cancer risk, with adjustment for age at baseline interview, year of interview, dialect group, level of education, daily alcohol drinking, number of live births, age when menstrual periods became regular, and family history of breast cancer. Consumption of saturated, monounsaturated or polyunsaturated fat overall was unrelated to risk. On the other hand, high levels of dietary n-3 fatty acids from fish/shellfish (marine n-3 fatty acids) were significantly associated with reduced risk. Relative to the lowest quartile of intake, individuals in the higher three quartiles exhibited a 26% reduction in risk (relative risk (RR)=0.74, 95% confidence interval (CI)=0.58, 0.94)); RRs were similar across the top three quartiles of intake (0.75, 0.75, 0.72, respectively). Overall, there was no association between n-6 fatty acids and breast cancer risk. However, among subjects who consumed low levels of marine n-3 fatty acids (lowest quartile of intake), a statistically significant increase in risk was observed in individuals belonging to the highest *vs* the lowest quartile of n-6 fatty acid consumption (RR=1.87, 95% CI=1.06–3.27); the corresponding RR for advanced breast cancer was 2.45 (95% CI=1.20–4.97, *P* for trend=0.01). To our knowledge, these are the first prospective findings linking the intake of marine n-3 fatty acids to breast cancer protection.

The overall effect of dietary fat on breast cancer risk has been much studied with inconclusive results (Hunter *et al*, 1996; Smith-Warner *et al*, 2001). There is relatively sparse information on the risk of breast cancer and individual fatty acids, which have demonstrated differential or even opposing effects in experimental systems of mammary carcinogenesis (Fay *et al*, 1997). Accumulating evidence suggests that fish oil or the constituent n-3 fatty acids may exert a protective effect against breast cancer development. The protective effect has been observed in both the carcinogen-induced tumour model and transplantable tumour model (Rose and Connolly, 1999). On the other hand, animal experiments consistently show that n-6 fatty acid promotes mammary tumorigenesis (Fay *et al*, 1997). Experimental data (Karmali *et al*, 1984; Gabor and Abraham, 1986; Karmali, 1987; Rose and Connolly, 1993) also suggest that the tumour-enhancing effect of n-6 fatty acids can be abrogated by marine n-3 fatty acids.

Relatively sparse data are available on the potentially opposing and interactive effects of n-3 and n-6 fatty acids on breast cancer in humans (Simonsen *et al*, 1998; Maillard *et al*, 2002; Goodstine *et al*, 2003). Here, we report on the effects of different types of fatty acids (saturated, monounsaturated, polyunsaturated) and certain individual fatty acids (n-3, n-6) on breast cancer development in the Singapore Chinese Health Study, an ongoing prospective cohort study with a focus on diet and cancer risk. Relative to the better studied western populations, Singapore Chinese consume less dietary fat overall, but have one of the highest consumption rates for fish (Hankin *et al*, 2001). Although breast cancer incidence is historically low in Chinese women, breast cancer incidence in the Singapore Chinese has doubled between the 1970s and 1990s (Seow *et al*, 1996; Chia *et al*, 2000).

## MATERIALS AND METHODS

### Study population

The Singapore Chinese Health Study has been described previously (Hankin *et al*, 2001). Briefly, the cohort was drawn from permanent residents or citizens of Singapore living in government-built housing estates (where 86% of the Singapore population lived during the study enrollment period). The eligible age range for cohort enrollment was 45–74 years, and was restricted to the two major dialect groups of Chinese in Singapore, Hokkien, and Cantonese. Between April 1993 and December 1998, 63 257 subjects (about 85% of eligible subjects) were recruited, of whom 56% (*n*=35 298) were women. The 564 women who reported a history of cancer at baseline were excluded from the present study. The study was approved by the institutional review boards of the University of Southern California and the National University of Singapore.

### Baseline dietary assessment

At recruitment, an in-person interview was conducted in the subject's home by a trained interviewer, using a structured and validated, 165-item food frequency questionnaire assessing the usual intake pattern during the previous 12 months (Hankin *et al*, 2001). The questionnaire also requested information on demographics, lifetime use of tobacco (cigarettes, water-pipe), current physical activity, reproductive history (women only), occupational exposure, medical history, and family history of cancer.

The food frequency questionnaire listed 14 seafood items, including fresh fish (fish ball or cake, deep fried fish, pan or stir fried fish, boiled or steamed fish), fresh shellfish (shrimp or prawn, squid or cuttlefish), dried/salted fish (salted fish, ikan bilis, dried fish, other dried seafoods such as dried shrimp, dried oyster, dried cuttlefish), and canned fish (canned tuna, canned sardine). The average portion weight (without bone) for fresh fish was approximately 60 g and for fresh shellfish, dried/salted fish, and canned fish approximately 35, 10 g, and 60 g, respectively.

### Case ascertainment

Incident cancer cases and deaths among cohort members were identified through linkage of cohort files with databases of the nationwide Singapore Cancer Registry (Parkin *et al*, 2002) and the Singapore Registry of Births and Deaths. Migration out of Singapore, especially among housing estate residents, is negligible (Department of Statistics, Singapore Ministry of Trade and Industry, 2001). As of 31 December 2000 (an average of 5.3 years of follow-up), 314 female cohort participants who were free of cancer at baseline had developed breast cancer at an average age of 59.5 years. Of these, 95 were localised (*in situ* or stage I) and 188 advanced (119 stage II, 37 stage III, and 32 stage IV). In 31 cases, the stage was unknown due to lack of access to medical charts. In all, 93 patients were premenopausal at baseline, the remaining 221 postmenopausal.

### Data analysis

Person-years of follow-up were counted from the date of recruitment to the date of diagnosis of breast cancer, death, or 31 December 2000, whichever occurred first.

We examined the relationships of dietary total, saturated, monounsaturated, and polyunsaturated fat intakes with risk of breast cancer. We then separated polyunsaturated fatty acids into n-3 and n-6 fatty acids. n-3 fatty acids were further categorised by food sources: seafood (eicosapentaenoic acid (EPA) and docosahexaenoic acid (DHA)) *vs* other foods (alpha-linolenic acid). Levels of various dietary fats in individual subjects were computed from fat contents of food items listed in the Singapore Food Composition Table (Hankin *et al*, 2001). To adjust for energy intake, all food groups and nutrients were expressed in weight unit per 1000 kcal or percentage of total energy.

Proportional hazards regression methods (Cox, 1972) were used to examine the associations between dietary exposure levels and breast cancer risk, measured by relative risks (RRs) and their corresponding 95% confidence intervals (CIs) and *P*-values. Study subjects were grouped into quartiles based on the distribution of the entire female cohort (see Appendix A). The linear trend tests for exposure–disease associations were based on ordinal values of the quartiles (0, 1, 2, 3).

In all analyses, we adjusted for the following potential confounders: age at baseline interview (years), dialect group (Hokkiens or Cantonese), year of interview (1993–1998), level of education (no formal education, primary school only, secondary school or higher), daily alcohol drinker (yes, no), family history of breast cancer (yes, no), number of live births (0, 1–2, 3–4, 5+), and age when period became regular (⩽12, 13–14, 15–16, 17+ years or irregular). Inclusion of body mass index (BMI) (<20, 20–<24, 24–<28, or ⩾28 kg m^−2^) in multivariate models involving postmenopausal women only did not appreciably alter the fish– (marine n-3 fatty acids) breast cancer association. Therefore, all RRs related to postmenopausal women in this report were unadjusted for BMI.

Statistical computing was conducted using SAS version 8.2 (SAS Institute Inc., Cary, NC, USA) and Epilog windows version 1.0 (Epicenter Software, Pasadena, CA, USA) statistical software packages. All *P*-values quoted are two-sided.

## RESULTS

Factors positively associated with risk were level of education, BMI (in postmenopausal women), age at first birth, and family history of breast cancer. Factors inversely associated with risk were age when period became regular and number of live births (Koh *et al*, 2003).

Almost all women (99.7%) in this study reported consuming some seafood. On average, the mean fish/shellfish intake is 52 g/day. When women were grouped by intake of marine n-3 fatty acids in quartiles, those in the highest consumed, on average, 80 g of fish/shellfish per day, while those in the lowest had 25 g. Consumption of marine n-3 fatty acids was positively associated with the percentage of total energy from fat, saturated, monounsaturated, and polyunsaturated fatty acids, but inversely associated with alcohol consumption (Table 1

**Table 1:** Distribution of selected characteristics of cohort participants at baseline, according to intake levels of marine n-3 fatty acids, the Singapore Chinese Health Study

	**Dietary marine n-3 fatty acid in quartiles**			
	**1st**	**2nd**	**3rd**	**4th**
Mean fish/shellfish intake (g day^−1^)	24.5	44.2	58.3	80.5
Mean fresh fish/shellfish intake (g day^−1^)	22.9	41.8	55.5	77.1
Mean preserved fish/shellfish intake (g day^−1^)	1.6	2.4	2.8	3.4
Mean fish intake, fresh and preserved (g day^−1^)	21.3	39.6	53.2	75.0
Mean shellfish intake, fresh and preserved (g day^−1^)	3.2	4.6	5.1	5.5
				
No formal education (%)	40.4	39.9	39.2	42.6
Mean age (years)	57.1	56.2	56.0	55.9
Mean body mass index (kg m^−2^)	23.0	23.1	23.3	23.5
Daily alcohol drinker (%)	1.4	1.2	1.1	0.9
Current smoker (%)	6.8	6.0	5.6	6.7
Total energy (kcal day^−1^)	1391	1443	1419	1339
Total fat (%kcal)	23.5	25.3	26.3	27.4
Saturated fat (%kcal)	8.4	9.0	9.2	9.4
Monounsaturated fat (%kcal)	7.9	8.5	8.8	9.2
Polyunsaturated fat (%kcal)	4.8	5.1	5.5	5.8

).

Table 2

**Table 2:** Dietary intake levels of various fat components in relation to risk of breast cancer, the Singapore Chinese Health Study

	**Dietary intake level in quartiles**				
	**1st**	**2nd**	**3rd**	**4th**	***P* linear trend**
*Total fat*					
No. of cases	80	74	89	71	
RR (95% CI)	1.00	0.92 (0.67–1.27)	1.13 (0.83–1.53)	0.94 (0.68–1.31)	0.95
					
*Saturated fat*					
No. of cases	92	76	73	73	
RR (95% CI)	1.00	0.85 (0.63–1.16)	0.86 (0.63–1.18)	0.92 (0.67–1.26)	0.59
					
*Monounsaturated fat*					
No. of cases	72	93	80	69	
RR (95% CI)	1.00	1.29 (0.95–1.76)	1.13 (0.82–1.56)	1.02 (0.73–1.43)	0.90
					
*Polyunsaturated fat*					
No. of cases	69	86	60	99	
RR (95% CI)	1.00	1.24 (0.90–1.71)	0.83 (0.59–1.18)	1.27 (0.92–1.74)	0.46
					
*n-3 fatty acid*					
No. of cases	88	73	74	79	
RR (95% CI)	1.00	0.82 (0.60–1.12)	0.84 (0.62–1.15)	0.87 (0.64–1.18)	0.40
					
*n-3 fatty acid, marine*					
No. of cases	97	73	74	70	
RR (95% CI)	1.00	0.75 (0.55–1.01)	0.75 (0.55–1.02)	0.72 (0.53–0.98)	0.04
					
*n-3 fatty acid, other foods*					
No. of cases	82	73	73	86	
RR (95% CI)	1.00	0.88 (0.64–1.20)	0.89 (0.64–1.22)	1.00 (0.73–1.36)	0.97
					
*n-6 fatty acid*					
No. of cases	70	81	66	97	
RR (95% CI)	1.00	1.15 (0.84–1.59)	0.90 (0.64–1.26)	1.22 (0.89–1.67)	0.45

RRs were adjusted for age at baseline interview (years), year of recruitment (1993–1998), dialect group (Cantonese, Hokkien), education (no formal education, primary school, secondary school or higher), daily alcohol drinker (yes, no), family history of breast cancer (yes, no), age when period became regular (⩽12, 13–14, 15–16, 17+ years or irregular), and number of live births (0, 1–2, 3–4, 5+).

presents the intake of total and subtypes of fatty acids in relation to risk. High levels of dietary n-3 fatty acids from fish/shellfish (marine n-3 fatty acids) were significantly associated with reduced risk. Relative to the lowest quartile of intake, individuals in the top three quartiles exhibited a 26% reduction in risk (RR=0.74, 95% CI=0.58, 0.94); RRs were similar across the top three quartiles of intake (0.75, 0.75, 0.72, respectively). On the other hand, no relation between n-3 fatty acids from other foods and risk was noted, nor with any other type of fatty acid including total, saturated, monounsaturated, polyunsaturated, and n-6 fatty acids (Table 2).

The inverse relationship between marine n-3 fatty acids and breast cancer risk was mainly confined to postmenopausal women and those with advanced disease; similar results were obtained for fish/shellfish intake (Table 3

**Table 3:** Dietary intake levels of fish and marine n-3 fatty acid in relation to risk of breast cancer by stage of disease and menopausal status, the Singapore Chinese Health Study

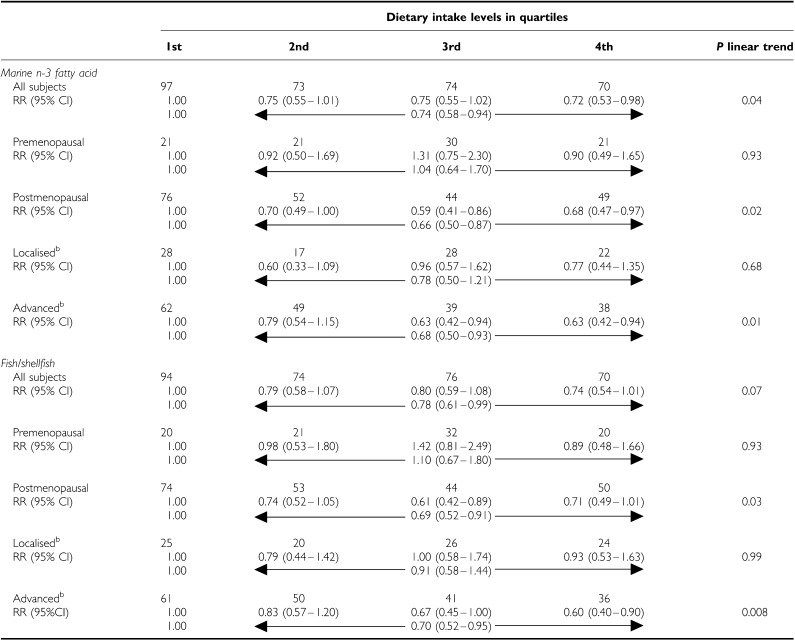					

a RRs were adjusted for age at baseline interview (years), year of recruitment (1993–1998), dialect group (Cantonese, Hokkien), education (no formal education, primary school, secondary school or higher), daily alcohol drinker (yes, no), family history of breast cancer (yes, no), age when period became regular (⩽12, 13–14,15–16, 17+ years or irregular), and number of live births (0, 1–2, 3–4, 5+).

^b^The sum was less than the total number of cases due to exclusion of cases with unknown stage in these analyses.

).

Prompted by experimental findings, we examined the n-6 fatty acid–breast cancer relationship stratified by dietary marine n-3 fatty acids. Among subjects in the lowest quartile of intake of marine n-3 fatty acids, increasing levels of n-6 fatty acids were significantly associated with increased risk, particularly among advanced breast cancer cases (*P* for trend=0.01) (Table 4

**Table 4:** Consumption levels of n-6 fatty acids in relation to risk of breast cancer by stage of disease and consumption levels of marine n-3 fatty acids (The Singapore Chinese Health Study)

					**Stage of disease^a^**			
			**All cases**		**Localised**		**Advanced**	
**Marine n-3 fatty acids**	**n-6 fatty acids (quartiles)**	**Person-years**	**No.**	**RR (95% CI)^b^**	**No.**	**RR (95% CI)^b^**	**No.**	**RR (95% CI)^b^**
1st quartile								
	1st	15 371	25	1.00	8	1.00	14	1.00
	2nd	10 630	27	1.53 (0.88–2.63)	10	1.74 (0.68–4.42)	14	1.41 (0.67–2.97)
	3rd	10 232	19	1.12 (0.61–2.05)	3	0.52 (0.14–1.97)	15	1.59 (0.76–3.33)
	4th	8541	26	1.87 (1.06–3.27)	7	1.52 (0.54–4.27)	19	2.45 (1.20–4.97)
	*P* for trend			0.08		0.88		0.01
								
*2nd–4th quartiles*								
	1st	30 314	45	1.00	12	1.00	28	1.00
	2nd	34 542	54	1.03 (0.69–1.53)	14	0.95 (0.44–2.06)	33	1.03 (0.62–1.71)
	3rd	35 850	47	0.83 (0.55–1.26)	17	1.05 (0.50–2.22)	24	0.69 (0.40–1.21)
	4th	39 585	71	1.08 (0.73–1.58)	24	1.29 (0.63–2.62)	41	1.00 (0.61–1.64)
	*P* for trend			0.88		0.40		0.73

a The sum is less than the total number of cases due to exclusion of cases with unknown stages in these analyses.

b Adjusted for age at baseline interview (years), year of recruitment (1993–1998), dialect group (Cantonese, Hokkien), education (no formal education, primary school, secondary school or higher), daily alcohol drinker (yes, no), family history of breast cancer (yes, no), age when period became regular (⩽12, 13–14, 15–16, 17+ years or irregular), and number of live births (0, 1–2, 3–4, 5+).

). The n-6–breast cancer association was not affected by menopausal status. There was no association between n-6 fatty acids and risk in the higher three quartiles of intake of marine n-3 fatty acids (Table 4).

Since consumption of seafood/n-3 fatty acid may be a marker of other dietary factors that exert beneficial effects on breast cancer, we examined whether the presence of other dietary factors could explain the observed inverse association shown by marine n-3 fatty acids. The inverse association remained after dietary factors including the total dietary fibre, vitamin A, and total carotenoids were individually adjusted for (data not shown).

In seven of the 314 cases, there was a family history of breast cancer and five of these belonged to the lowest quartile of intake of marine n-3 fatty acids, yielding an RR of 4.2 (95% CI=1.7–10.4) for a family history of breast cancer in this dietary subgroup. The corresponding RR (95% CI) among those in the higher three quartiles of intake of marine n-3 fatty acids was 0.7 (0.2–2.9).

In order to explore the possibility that the observed inverse association between fish intake and breast cancer might be an artefact due to the decreased consumption of fish in subclinical cancer patients, we repeated all analyses after excluding all cancer occurrences and person-year counts during the first 2 years of follow-up. Results were comparable to those based on a complete follow-up of the entire cohort (data not shown).

In this study population, fish and shellfish account for roughly 30% of dietary n-3 fatty acid consumption. Other major sources of n-3 fatty acids are legumes (10%), grain products (22%), and cooking oils (10%). The major sources of n-6 fatty acids are meats (10%), grain products (20%), and cooking oils (40%).

## DISCUSSION

The present study found no evidence of an association between breast cancer and total, saturated, monounsaturated, or polyunsaturated dietary fat intake, in agreement with previous studies, including a meta-analysis of seven epidemiologic cohort studies from four countries totalling more than 330 000 women and nearly 5000 incident cases of breast cancer, as well as more recent analysis (Hunter *et al*, 1996; Harrison and Waterbor, 1999; Smith-Warner *et al*, 2001).

High levels of dietary marine n-3 fatty acids were significantly associated with a reduced risk of breast cancer in the present study. Relative to the lowest quartile of intake, individuals in the top three quartiles exhibited a 26% reduction in risk. These observations are consistent with the available experimental evidence. Marine n-3 fatty acids inhibit chemically induced mammary tumours in rats, and transplanted mammary tumours in rats and mice (Rose and Connolly, 1999). They also were shown to retard the growth and metastasis of human breast cancer cells in nude mice (Rose and Connolly, 1999).

Ecological studies support the notion that high consumption of fish is associated with low incidence of breast cancer (Rose, 1997, Terry *et al*, 2003). Coastal- and rural-dwelling Japanese and Eskimos, who traditionally consume large quantities of marine n-3 fatty acids, have low breast cancer rates (Rose, 1997).

Although intake of marine n-3 fatty acids was rarely calculated, fish consumption and its relation with breast cancer has been reported in many studies (reviewed in Terry *et al*, 2003). At least 21 case–control studies have examined this relationship (Franceschi *et al*, 1995; Goodstine *et al*, 2003; Terry *et al*, 2003). Only eight of these studies found a protective effect. Several case–control studies have examined marine n-3 fatty acids in the adipose tissue between cases and controls. Although these specimens were obtained postcancer diagnosis in cases, the concern of dietary changes in cases, as a consequence of clinical symptoms, had been partially addressed by the fact that the half-life of fatty acids in adipose tissue is in the order of 600 days (Beynen *et al*, 1980). Results of these latter studies are mixed. Some (Zhu *et al*, 1995; Maillard *et al*, 2002) but not all (London *et al*, 1993; Petrek *et al*, 1994; Simonsen *et al*, 1998) studies found a protective effect.

Six cohort studies, in Norway (Vatten *et al*, 1990), Japan (Key *et al*, 1999), and in the US (Stampfer *et al*, 1987; Toniolo *et al*, 1994; Gertig *et al*, 1999; Holmes *et al*, 1999), have investigated fish or marine n-3 fatty acid intake. In the study from Norway involving 152 breast cancer cases (Vatten *et al*, 1990), no association was found between the overall intake frequency and risk, although an inverse relation with the frequency of main meals containing poached fish was observed. In the Japanese study (Key *et al*, 1999), ⩾5 servings of dried fish/week was associated with a 50% lower risk than women who consumed ⩽1 serving/week (*P* for trend=0.03). No association was found in the four studies from the US (Stampfer *et al*, 1987; Toniolo *et al*, 1994; Gertig *et al*, 1999; Holmes *et al*, 1999). Four cohort studies have explored the marine n-3 fatty acid–breast cancer relationship using biomarkers (prediagnostic serum phospholipids or erythrocyte membranes) (Vatten *et al*, 1993; Chajes *et al*, 1999; Pala *et al*, 2001; Saadatian-Elahi *et al*, 2002). Only one of these four studies reported an inverse association between high levels of DHA measured in erythrocyte membranes and breast cancer (*P* for trend=0.05) (Pala *et al*, 2001). The discrepancies among cohort studies may be due to differences in ranges of intake across the various populations. The two studies that showed an inverse association were in high-consumption countries (Hursting *et al*, 1990). In contrast, intake levels of fish are low in the US; only 15% of US women eat more than one serving of fish per week (Gillum *et al*, 1996).

Overall, there was no association between n-6 fatty acids and breast cancer in the present study. However, among women consuming low levels of marine n-3 fatty acids, high intake of n-6 fatty acids was associated with increased risk, consistent with the experimental evidence that the stimulatory effect of n-6 fatty acid in mammary carcinogenesis depends on the background levels of marine n-3 fatty acids (Karmali *et al*, 1984; Gabor and Abraham, 1986; Rose and Connolly, 1993). Both types of fatty acids are substrates for human eicosanoid production, share the same enzymes for the synthesis of prostaglandins and leukotrienes, and compete for each other at the cyclooxygenase level (Bartsch *et al*, 1999). The metabolism of n-6 fatty acids is mediated by three major enzymatic pathways: the cyclooxygenase, the lipoxygenase and cytochrome *P*-450 epoxygenase pathways, producing prostaglandins, leukotrienes, and hydroxyl-eicosatetraenoic and epoxy-eicosatrienoic acids. It has been suggested that n-6 fatty acids promote breast cancer tumorigenesis and tumour cell proliferation directly and indirectly via increased synthesis of these cyclooxygenase- and lipooxygenase-catalysed products (Noguchi *et al*, 1995).

In the present study, the positive association between the family history of breast cancer and personal risk was especially pronounced among women with low intake of fish. Germline mutation in the putative tumour-suppressor gene BRCA1 is believed to account for close to half of familial breast cancers (Rosen *et al*, 2003). It has been shown that BRCA1 mRNA expression levels are the lowest in tumours of mutation carriers, intermediate in tumours of sporadic cancers, and highest in the surrounding normal tissues from either mutation carriers or sporadic cancers (Kainu *et al*, 1996). Interestingly, treatment with the two main marine n-3 fatty acids (EPA and DHA) was shown to increase BRCA1 mRNA expressions in breast cancer cell lines (Bernard-Gallon *et al*, 2002). Our results, although based on a small number of subjects, suggest that marine n-3 fatty acids may be a therapeutic option in women with a BRCA1 mutation.

Reasons for the stronger associations in postmenopausal women and those with advanced stage are not clear. Since only 30% of the cases included in our study were diagnosed in premenopausal women, the weaker associations in this subgroup may be a chance finding due to small numbers. However, one study (Zhu *et al*, 1995) found significantly lower levels of DHA in breast adipose tissue only in postmenopausal patients compared to controls with benign breast disease. Similarly, the stronger associations in advanced cases of breast cancer may be a chance finding. On the other hand, low DHA levels in breast adipose tissues were predictive of metastasis with borderline statistical significance (Bougnoux *et al*, 1994).

This study has several strengths. First, exposure assessment preceded cancer diagnosis; so recall bias is not a concern. In addition, the dietary information was solicited in-person with the aid of frequency charts and food photographs, which should be of higher quality than those obtained via self-administered questionnaires. Finally, our baseline food frequency questionnaire has been validated against a series of 24-h recalls (Hankin *et al*, 2001) as well as two urinary biomarkers of nutrients (Seow *et al*, 1998a, 1998b).

In conclusion, this is the first set of prospective results linking the intake of marine n-3 fatty acids to breast cancer protection. Our observations may have practical implications in prevention and treatment strategies for breast cancer, suggesting that an intake level of approximately 40 g of fish/shellfish per day (median value of second quartile of intake) can reduce breast cancer risk by 25%. Finally, consistent with experimental results, our data suggest that high consumption of n-6 fatty acids was associated with increased risk of breast cancer among women consuming low levels of marine n-3 fatty acids.

## Appendix A

The quartile cutpoints for various dietary fats in the Singapore Chinese Health Study are given in Table A1

**Table A1:** Quartile cutpoints for various dietary fats, the Singapore Chinese Health Study

	**Quartile of intake**			
	**1st**	**2nd**	**3rd**	**4th**
Total fat (% kcal)	⩽21.87	21.88–25.64	25.65–29.43	⩾29.44
Saturated fat (% kcal)	⩽7.18	7.19–8.90	8.91–10.72	⩾10.73
Monounsaturated fat (% kcal)	⩽7.23	7.24–8.58	8.59–9.99	⩾10.00
Polyunsaturated fat (% kcal)	⩽3.95	3.96–4.90	4.91–6.26	⩾6.27
n-3 fatty acid (% Kcal)	⩽0.43	0.44–0.51	0.52–0.60	⩾0.61
n-3 fatty acid, marine (% kcal)	⩽0.13	0.14–0.19	0.20–0.25	⩾0.26
n-3 fatty acid, other foods (% kcal)	⩽0.26	0.27–0.31	0.32–0.37	⩾0.38
n-6 fatty acid (% kcal)	⩽3.45	3.46–4.34	4.35–5.66	⩾5.67
Fish/shellfish (g 1000 kcal^−1^)	⩽25.07	25.08–35.52	35.53–47.65	⩾47.66

.
